# Dementia Risk in Irradiated Patients With Head and Neck Cancer

**DOI:** 10.1097/MD.0000000000001983

**Published:** 2015-11-13

**Authors:** Jin-Hua Chen, Yu-Chun Yen, Shing-Hwa Liu, Fei-Peng Lee, Kuan-Chou Lin, Ming-Tang Lai, Chia-Che Wu, Tsung-Ming Chen, Sheng-Po Yuan, Chia-Lun Chang, Szu-Yuan Wu

**Affiliations:** From the Biostatistics Center and School of Public Health, Taipei Medical University (J-HC, Y-CY); Institute of Toxicology, College of Medicine, National Taiwan University (S-HL, S-YW); Department of Otorhinolaryngology (F-PL, M-TL, C-CW, T-MC, S-PY); Department of Oral and Maxillofacial Surgery (K-CL); Department of Hemato-Oncology (C-LC); Department of Radiation Oncology, Wan Fang Hospital, Taipei Medical University (S-YW); Department of Internal Medicine, School of Medicine, College of Medicine, Taipei Medical University, Taipei (S-YW); and Department of Biotechnology, Hungkuang University, Taichung, Taiwan, Republic of China (S-YW).

## Abstract

Patients with head and neck cancer are treated through surgery, radiotherapy (RT), and chemotherapy (CT). Carotid artery damage and neurotoxicity were previously observed in these patients. This study estimated the dementia risk associated with different treatment modalities in a head and neck cancer population with long-term follow-up.

Taiwan's National Health Insurance claims database and a cancer registry database from the Collaboration Center of Health Information Application were linked for the present analysis. Patients with head and neck cancer, treated from January 1, 2002 to December 31, 2010, were included in the study. The follow-up duration was the period from the index date to December 31, 2012. Inclusion criteria were head and neck cancer; an age >20 years; and having undergone surgery, CT, concurrent CT, or surgery with adjuvant treatment. Exclusion criteria were another cancer diagnosed before the head and neck cancer, death or being diagnosed with dementia within 2 years after the treatment of the head and neck cancer, stroke before the index date, distant metastasis, in situ carcinoma, sarcoma, head and neck cancer recurrence, an unknown sex, and an age <20 years. In total, 20,135 patients were included.

In patient groups that underwent surgery alone, surgery and adjuvant chemoradiotherapy, and chemoradiotherapy alone, the dementia incidence per 1000 person-years was 1.44, 1.04, and 1.98, respectively. The crude hazard ratio (HR) of dementia was 1.84 (95% confidence interval [CI] 1.21–2.81) in the RT with or without CT group. After adjustment for age, sex, clinical stage, and comorbidity, the HR was 1.92 (95% CI 1.14–3.24). Examining the dementia risk in patients who received different treatment modalities according to the Cox proportional-hazard model revealed that an age >65 years and having undergone RT with or without CT were risk factors (*P* < 0.001 and *P* = 0.015; and HRs of 16.5 and 1.92, respectively). The dementia risk in patients at different clinical stages was not significantly different among the various treatment groups, regardless of whether the patients received RT. However, younger (<65 y) patients who received RT with or without CT had a 2.96-fold (95% CI 1.24–7.08) higher risk of dementia and a 3.54-fold (95% CI 1.32–9.51) higher adjusted HR compared with the surgery-alone group. Patients who received a total radiation dose >6660 cGy exhibited a 1.69-fold (95% CI 0.97–2.95, *P* = 0.063) higher dementia risk compared with those who received a total radiation dose <6660 cGy.

Receiving a higher radiation dose increased the dementia risk and persistently escalated the dementia incidence even 9 years after RT. Younger (<65 y) patients have a high risk of dementia after RT. The selection of young patients for dose de-escalation requires improvement for reducing irradiation to the neck and areas near brain tissues, particularly in Taiwan, where the median patient age is 53 years.

## Abstract 2

Supplemental Digital Content is available in the text

## INTRODUCTION

Head and neck cancer is the 4th leading cause of cancer deaths and the 6th most common cancer in Taiwan according to the Taiwan Cancer Registry report, 2011 edition, published on the website of the Health Promotion Administration, Ministry of Health and Welfare.^1^ Most Taiwanese patients with head and neck cancer are men with a betel nut-chewing habit and median age of 53 years, and may still be a crucial economic contributor to their families.^1,2^ Surgery, radiotherapy (RT), and chemotherapy (CT) are essential treatments for patients with head and neck cancer.^3^ Therefore, the possible side effects of different treatment modalities demand immediate attention.

Radiotherapy plays a critical role in treating patients with head and neck cancer^3,4^; however, its long-term outcomes remain unsatisfactory. Some patients with long-term survival might experience unexpected late complications with a longer survival time. Previous studies have shown an increased risk of ischemic stroke after RT to the neck in patients aged <60 years.^5^ Some studies have reported that RT can have deleterious effects on cognitive function.^6,7^ The most common feelings that families and caregivers experience after dementia diagnosis are guilt, grief, loss, and anger. The effects on family, friends, and society are large, and health resource consumption is substantial.^8,9^

As per our review of relevant literature, no study has investigated the incidence of dementia after the initial treatment of head and neck cancer. We aimed to determine this incidence in patients with head and neck cancer, identified from Taiwan's National Health Insurance (NHI) database and a cancer registry database from the Collaboration Center of Health Information Application (CCHIA). These nationwide population-based databases enabled us to trace the medical service utilization history of all citizens and provided a unique opportunity for comparing the dementia risk between irradiated and nonirradiated patients with head and neck cancer, leading to an understanding of the relative hazards among different treatment modalities.

## MATERIALS AND METHODS

**FIGURE 1:**
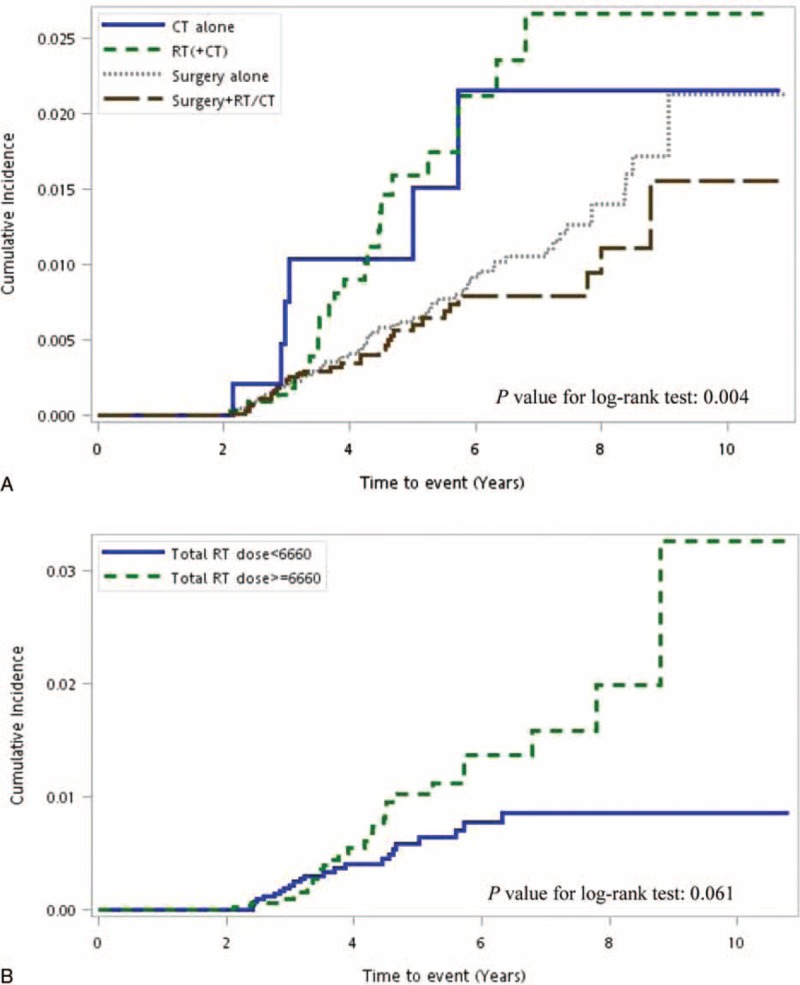
A, Cumulative incidence of dementia with different treatment modalities. B, Cumulative incidence of dementia in groups with varying total radiation doses.

Two cohorts from Taiwan's National Health Insurance (NHI) database and the CCHIA cancer registry database were combined for the present analysis. Both databases covered approximately 99% of the Taiwanese population. Patients with head and neck cancer from January 1, 2002 to December 31, 2010 were included in the study. The follow-up duration was the period from the index date to December 31, 2012. Taiwan's NHI Bureau has released research-oriented data sets through the CCHIA; these data sets include all original claims data and registration files for beneficiaries enrolled under the CCHIA. Taiwan launched the CCHIA program in 1995, and 99% of the Taiwanese population was covered in 2012. Therefore, the CCHIA enables researchers to trace all medical services utilized by patients. The cancer registry database includes information on the clinical stage, treatment modalities, pathological data, CT regimen, concurrent and sequential CT/RT, and RT doses. The diagnoses of included patients were pathologically confirmed, and patients newly diagnosed with head and neck cancer had no other cancers or distant metastasis. The data sets from the NHI and cancer registry databases include complete information on medical care behaviors, costs, medical institutions, and physicians for all inpatients and outpatients enrolled in the NHI. Before accessing the data sets, researchers must sign an agreement contract for protecting patient information. Researchers are permitted to access the CCHIA database to analyze specific topics. Patient identification numbers in the data sets are encrypted, preventing the identification of a specific patient. The inclusion criteria were head and neck cancer (identified according to the International Classification of Diseases, Ninth Revision, Clinical Modification [ICD-9-CM] 140.0–148.9); an age >20 years; and having undergone surgery, CT, concurrent CT, or surgery with adjuvant treatment. Furthermore, for all patients, the index date was considered the beginning of the first treatment, such as surgery, RT, or CT. The exclusion criteria were prior cancer, death or been diagnosed with dementia within 2 years after the cancer treatment, stroke before the index date, distant metastasis, in situ carcinoma, sarcoma, head and neck cancer recurrence, an unknown sex, and an age <20 years. No patient who had undergone surgery with adjuvant therapy not including RT/CT was included in our study. In total, 20,135 patients were included. We divided different treatment modalities into different arms for comparing their outcomes. Arm 1 comprised patients who received surgery alone. Arm 2 comprised patients who received surgery with adjuvant RT, surgery with adjuvant CT, or surgery with concurrent CT/RT (surgery + RT/CT). Arm 3 consisted of patients who received concurrent CT/RT without surgery or RT alone (RT/CT). We further divided arm 3 into a group of patients receiving CT alone (arm 3–1) and a group of patients receiving RT ± CT, RT alone, or concurrent chemoradiotherapy (CCRT; arm 3–2) to clarify the effect of RT (Tables 1 and 2, Figure 1A, and Supplemental Table 1, http://links.lww.com/MD/A507). The end point was the incidence of dementia (ICD-9-CM 331.0, 290.4, 290.0–290.3) among different treatment modalities, with arm 1 serving as the reference group.

**TABLE 1:**
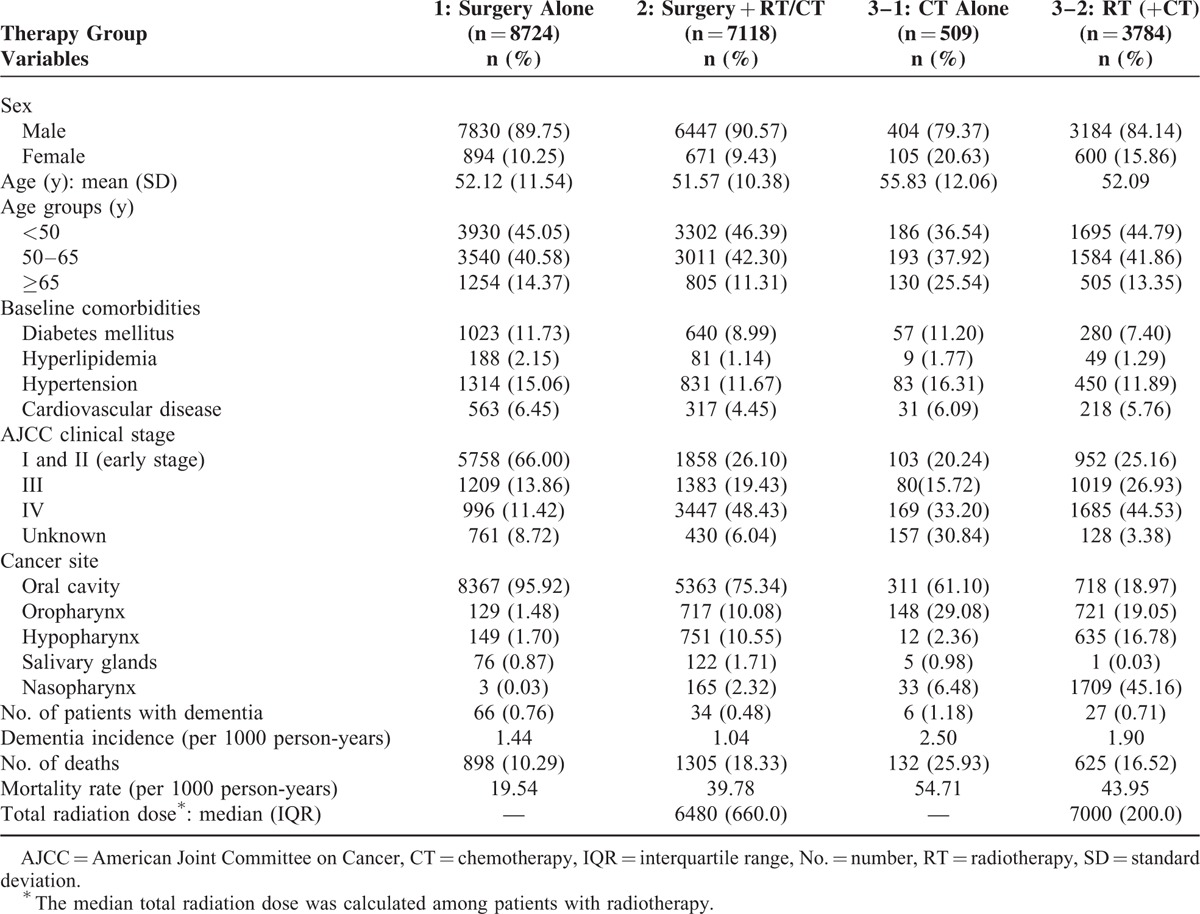
Baseline Characteristics and Follow-up Status of Patients With Different Treatment Modalities

**TABLE 2:**
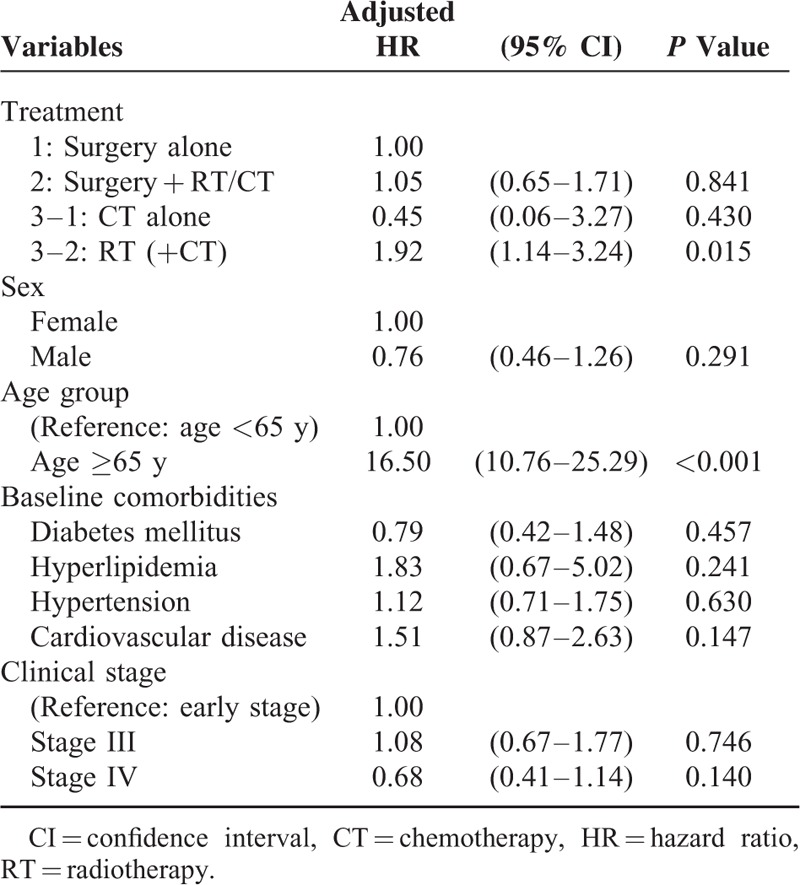
Multivariate Cox Proportional-hazard Model for the Risk of Dementia

The possible confounding factors for comorbidities included cerebrovascular disease (ICD-9-CM 430–432, 433–438), cardiovascular disease (ICD-9-CM 393–398, 410–414, 420–429, 440–449, 451–459; ICD-9-CM procedure codes 36.0, 36.01, 36.02, 36.05, 36.06, 36.1, 36.10–36.19, 391), hypertension (ICD-9-CM 401–405), hyperlipidemia (ICD-9-CM 272.0–272.4), and diabetes (ICD-9-CM 250). Comorbidities observed within 6 months before and after the index date were identified according to the main diagnosis code for the first admission or more than 2 main diagnosis codes for outpatient visits. Age, sex, and the clinical cancer stage according to the American Joint Committee on Cancer (AJCC) were adjusted for or stratified in the analysis. We also investigated the association of the radiation dose effect with the dementia incidence. We determined whether the time-dependent effect of dementia reaches a plateau or increases with time after irradiation to the head and neck areas.

The cumulative dementia incidence was estimated using the Kaplan–Meier method, and differences among treatment modalities and radiation doses were compared using the log-rank test. The Cox proportional-hazard regression model was used for calculating the hazard ratios (HRs) of dementia among patients who received different treatment modalities or radiation doses. HRs were adjusted for age, sex, baseline comorbidity, and clinical stage in the multivariate analysis. A stratified analysis was conducted for evaluating the dementia risk between different modalities for a similar age or clinical stage status. All analyses were conducted using SAS software Version 9.3 (SAS, Cary, NC). A 2-tailed *P* value <0.05 was considered significant.

## RESULTS

In total, 20,135 patients with head and neck cancer were included in the study, and the median follow-up duration was 4.18 (interquartile range 3.25) years. Arm 1 comprised 8724 patients, arm 2 comprised 7118 patients, arm 3–1 consisted of 509 patients, and arm 3–2 comprised 3784 patients (Table 1). The variables mentioned in the “Materials and Methods” section were similar in the 3 study arms, with the AJCC clinical cancer stage being prominently different. A higher percentage of patients with early-stage cancer underwent surgery alone, whereas patients with advanced-stage cancer were more often treated with surgery + RT/CT or RT/CT alone rather than with surgery alone. Most head and neck cancers were oral cavity cancers in the surgery-alone and surgery + RT/CT groups, but the proportion was relatively smaller in the RT/CT group. Nasopharyngeal cancer was the most prevalent cancer in the RT/CT group. Radiation doses were higher in the RT/CT group than in the other groups; median doses in the surgery + RT/CT and RT/CT groups were 6480 and 7000 cGy, respectively. These phenomena are reasonable and similar to those observed in our clinical practice. Detailed demographic characteristics of all patients are provided in Table 1.

In the surgery-alone, surgery + RT/CT, and RT/CT groups, the dementia incidence was 1.44, 1.04, and 1.98 per 1000 person-years, respectively. Mortality rates per person-years were 19.54, 39.78, and 45.51 in the 3 groups. We used arm 1 as the control arm for investigating the dementia risk after RT. The crude HRs for dementia were 1.84 (95% confidence interval [CI] 1.21–2.81) and 0.80 (95% CI 0.53–1.22) for arms 3 and 2, respectively. After adjustment for age, sex, clinical stage, and comorbidity, the HRs were 1.92 (95% CI 1.14–3.24) for arm 3–2 (RT ± CT) and 1.05 (95% CI 0.65–1.71) for arm 2. Figure 1A presents the cumulative dementia incidence for the 3 treatment arms; the dementia risk was higher in arm 3–2 (log-rank test, *P* = 0.004), and it seemed to plateau 7 years after RT. The Cox proportional-hazards regression model used for assessing the dementia risk in all patients revealed that an age >65 years and the receipt of RT ± CT without surgery were independent risk factors (*P* < 0.001 and *P* = 0.049, HRs 16.46 and 1.92, respectively; Table 2).

Clinical stages were analyzed through stratification (Table 3), and no significant differences were observed among the 3 treatment modalities, regardless of whether patients received RT. However, younger (<65 y) patients who received RT with or without CT (RT/CT) showed a 2.96-fold (95% CI 1.24–7.08) higher risk of dementia with a 3.54-fold (95% CI 1.32–9.51) higher adjusted HR compared with the surgery-alone group.

**TABLE 3:**
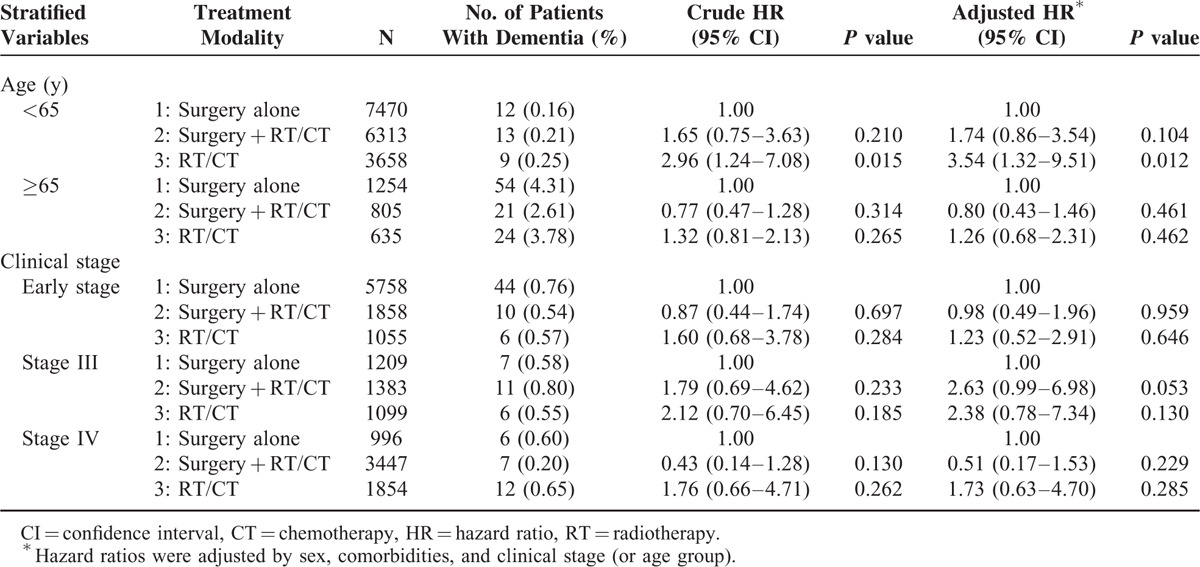
Stratified Cox Proportional-hazard Model for the Risk of Dementia and the Associated Treatment Modality

In addition to age, the effect of the radiation dose on the dementia risk was evaluated. The radiation doses administered to all patients are shown in Table 4, and the total radiation dose had a possible effect on the dementia risk. Table 4 shows that a total radiation dose >6660 cGy increased the dementia risk in all patients by 1.69-fold (95% CI 0.97–2.95, *P* = 0.063) relative to a total radiation dose <6660 cGy. Figure 1B shows the cumulative dementia incidence for different RT doses. A higher radiation dose resulted in a higher dementia risk (log-rank test, *P* = 0.0606), and the dose effect persisted even 9 years after RT. Contrastingly, a low dose <6660 cGy was associated with a low risk of dementia and possibly reached a plateau 7 years after RT. The dementia incidence per 1000 person-years was 1.388 and 0.995 in the 2 radiation dose groups.

**TABLE 4:**
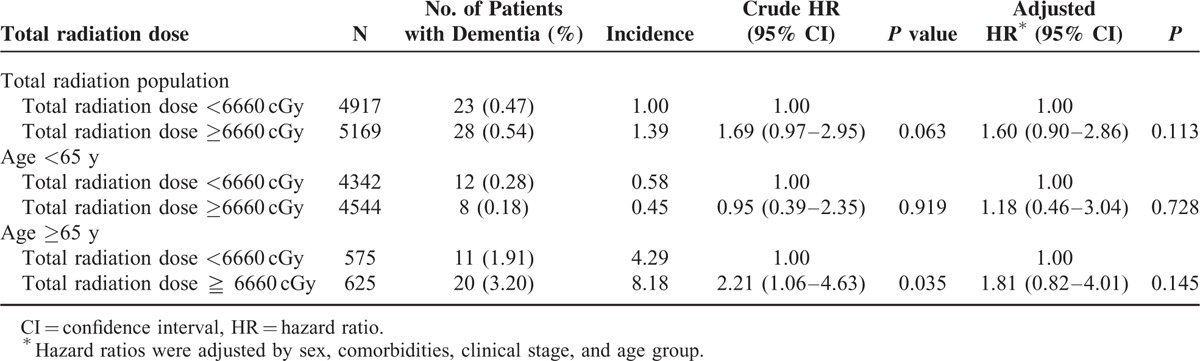
The Association Between the Incidence of Dementia and the Total Radiation Dose

## DISCUSSION

Regardless of the type, such as vascular dementia, multi-infarct dementia, and Alzheimer disease, dementia is caused by an irregular blood supply to the brain or other cardiovascular risk factors.^10–12^ Avoiding irradiation to the carotid arteries is extremely difficult in patients with head and neck cancer because of the high risk of neck failure and frequent malignant lymphadenopathy.^13–16^ Experimental animal studies have firmly established a causal relationship between irradiation and vascular disease. No changes were observed in the atherosclerotic lesions of out-of-field arteries, consistent with a local rather than a systemic effect of radiation. Macrophage accumulation was substantial around irradiated arteries after irradiation for 22 to 34 weeks. Intraplaque hemorrhage was limited at irradiated arteries.^17–20^ In vitro and in vivo experimental studies have indicated that RT causes acute upregulation of proinflammatory cytokines and adhesion molecules in the endothelium that recruit inflammatory cells to vascular injury sites.^21^ Furthermore, the induction of chronic oxidative stress is increasingly implicated in radiation-induced late tissue injury.^22^ The aforementioned findings are compatible with our clinical findings on the latent onset of dementia 3 years after RT and a time-dependent increase in the incidence even 7 years after RT (Fig. 1A).

Radiation-induced atherosclerotic progression to the carotid artery is associated with fibrosis formation in the intima–media layer, endothelial damage, and atheromatous plaques. Radiation-induced damage to the carotid artery is increased by occlusive changes in the vasa vasorum, leading to radiation-related ischemia of the arterial wall.^5,23–26^ These cardiovascular risk factors might contribute to additional risks of dementia in irradiated head and neck cancer patients.

According to our review of relevant literature, this study is the first to evaluate the dementia incidence in patients with head and neck cancer who received radiation therapies different from those involved in whole-brain RT. Previous studies have reported the development of dementia after the treatment of brain tumors or brain metastasis with RT administered alone or combined with CT, but the sample sizes were small.^27,28^ Moreover, the fraction size and total radiation dose investigated in our study differed from those administered during whole-brain RT or for treating brain tumors. Brain metastasis or tumors also induced dementia, even without treatment, when the tumors were located near the hippocampus. This study can draw attention to the experiences of and dementia incidence in irradiated head and neck cancer patients; the development of dementia in 20,135 patients who received single, bi, and trimodality therapies is also reported. The dementia incidence was relatively low, and the survival benefits experienced by irradiated head and neck cancer patients outweighed the harmfulness of dementia.

To our knowledge, this is the first study to investigate the association of RT administered to patients with head and neck cancer with dementia risk. The radiation dose effect, time effect, and dementia risk were positively correlated. The radiation dose is extremely critical for cancer control with a dose–effect association,^29,30^ although in our study, higher radiation doses (≥6660 cGy) resulted in a higher dementia risk (Table 4). The dementia incidence per 1000 person-years was 1.388 and 0.995 in the RT/CT and surgery + RT/CT groups, respectively. The absolute incidence of dementia was still low, and a larger sample size of patients with dementia was necessary for reaching a significant level. Dementia risk showed a dose–effect trend in Table 4 and Figure 1B. Understanding the risk of radiation-related dementia and the high risk of RT-induced dementia in patients with head and neck cancer is crucial. In our study, a radiation dose >6660 cGy increased the dementia risk, and the incidence was persistently higher with time after RT even after 9 years. At a dose <6660 cGy, the dementia risk reached a plateau within a shorter interval after RT, presumably because radiation-related vessel damage generally occurs within a longer time after RT.^31–33^ RT induces a continuum of vascular disease that begins with mild asymptomatic vascular thickening and progresses to severe vessel fibrosis with a dose–volume effect.^34–36^

Similar radiation-related vessel injury studies of stroke incidence have shown that RT increased the stroke risk in young patients with head and neck cancer treated with RT or CT.^5,37–40^ Lee et al^37^ and Huang et al^38^ reported that irradiated young (<55 y) patients with head and neck cancer had a higher risk of stroke. Our data also showed that young patients (<65 y) with head and neck cancer who received RT had a higher risk of dementia (Table 4). Our outcomes suggest that radiation-related vascular damage occurred in irradiated head and neck cancer patients with a latent-onset dementia risk and radiation-related neurotoxicity. Irradiation of the brain parenchyma within the target volume in patients with head and neck cancer may cause extensive cancer of the skull base or orbital, ethmoid, or sphenoid sinuses; nasopharyngeal cancer; or neurological complications.^41^ Gavrilovic et al^7^ revealed that administering the same radiation technique and dose to older patients (>60 y) caused more and intolerable radiation-related neurotoxicity, which was defined as a progressive neurological or cognitive impairment. Those outcomes may be compatible with ours; administering radiation at >6660 cGy to older patients (≥65 y) might cause an increase in radiation-related neurotoxicity (Table 4). Radiation-induced cognitive impairment is expected in long-term irradiated head and neck cancer patients with RT administered to brain tissues. Cheung et al^42^ reported that brain necrosis predicted an increase in cognitive impairment in 50 irradiated nasopharyngeal cancer patients who were longitudinally followed with neuropsychological testing. Furthermore, Hsiao et al^6^ reported that the cognitive outcome was poorer in patients with nasopharyngeal cancer treated with intensity-modulated RT when >10% of their temporal lobe volume received a total fractionated dose >6000 cGy compared with patients who received <6000 cGy. Our outcomes also confirmed the findings of previous studies indicating that radiation-related neurotoxicity was more severe in older patients (>65 y) and in those who received a high dose (>6660 cGy), with the crude HR being 2.21 (95% CI 1.06–4.63, *P* = 0.035; Table 4, Fig. 1B).

Our findings may influence the recommended treatment approach for younger patients (<65 y) with head and neck cancer. Administering a suitable radiation dose (<6660 cGy) for patients with head and neck cancer is vital. Young patients might have the potential for long-term survival after cancer treatment, and the survival time is sufficient for developing radiation-related dementia. Particularly in Taiwan, the median age of patients with head and neck cancer was 53 years, and most of these patients were aged <65 years. For instance, low-dose radiation (5400 cGy) is sufficient for treating some human papilloma virus-positive head and neck cancers according to a phase 2 ECOG 1308 study.^43^ These outcomes suggest that patient selection for dose de-escalation requires improvement.

The limitations of our study are that the actual radiation dose and volume delivered to the carotid artery and brain tissues could not be measured; therefore, the actual dose–volume effect is unclear. Although irradiation for patients with head and neck cancer sometimes involves neck and partial brain tissues, the maximum radiation dose point and irradiated target volume remain major unresolved problems. Therefore, a large randomized trial with a suitable regimen administered to well selected patients and comparing standard approaches is required for resolving the aforementioned problems. Moreover, dementia and other comorbidities were identified according to ICD-9-CM codes. However, Taiwan's NHI Bureau randomly reviews charts and interviews patients to verify the diagnostic accuracy. If any inconsistencies or malpractices, such as outlying charges or practices, are discovered, hospitals are investigated and heavily penalized. Finally, the database does not contain information about personal lifestyle, including tobacco and alcohol consumption, dietary habits, socioeconomic status, educational level, and the body mass index, which may also be risk factors for dementia. We did not analyze the outcomes of the treatment modalities listed in Supplemental Table 1 (http://links.lww.com/MD/A507) because of a small sample size of some groups; for instance, the sample size of the surgery + CT group was only 63 patients (data not shown). Taiwanese patients with head and neck cancer are treated postsurgically depending on their adverse outcome risks, including extracapsular nodal spread, positive margins, pT3 or pT4 primary tumor, N2 or N3 nodal disease, nodal disease at levels IV or V, perineural invasion, and vascular embolism. When patients are at a risk of only 1 minor outcome (pT3 or pT4 primary, N2 or N3 nodal disease, nodal disease at levels IV or V, perineural invasion, or vascular embolism), physicians administer adjuvant RT alone and not CT alone. However, when the risks are major (extracapsular nodal spread or positive margins) or patients are at a risk of 2 or more minor outcomes, adjuvant CCRT is typically administered. Most patients in arm 2 received surgery + RT or surgery with RT and CT, and the sample size of the surgery + CT group was comparatively small. Few patients postsurgically received adjuvant CT alone. As shown in Supplemental Table 1 (http://links.lww.com/MD/A507), although the sample size of arm 3–1 was small, we used this arm for determining the dementia incidence in patients who received single, bi, and trimodality therapies. The RT-alone group had the highest dementia incidence per 1000 person-years. We used a multivariate Cox proportional-hazards model for assessing the dementia risk and divided arm 3 into 3–1 (CT alone) and 3–2 (RT + CT). The dementia risk was significant in the RT ± CT group, but not in arm 3–1. Thus, RT is an independent risk factor for dementia. However, considering the magnitude and significance of the observed effects in this study, these limitations are unlikely to affect the conclusions.

## CONCLUSIONS

A high radiation dose results in a high risk of dementia in patients with head and neck cancer and persistently escalates the dementia incidence even 9 years after RT. Younger irradiated head and neck cancer patients had a high risk of dementia compared with the surgery-alone group. The selection of young patients with head and neck cancer for dose de-escalation requires improvement for preventing the delivery of unnecessarily high doses to the neck and areas near brain tissues.
